# Lightweight Architecture Optimization for Car Logo Detection Based on Visual State Space Models

**DOI:** 10.3390/s26216973

**Published:** 2026-11-04

**Authors:** Jingwen Gong, Junxing Zhang, Chunjuan Bo

**Affiliations:** 1 College of Information and Communication Engineering, Dalian Minzu University, Dalian 116600, China; 202412051032@stu.dlnu.edu.cn (J.G.); bcj@dlnu.edu.cn (C.B.); 2 College of Computer Science and Engineering, Dalian Minzu University, Dalian 116600, China

**Keywords:** car logo detection, state space model (SSM), lightweight object detection, Mamba-YOLO, dynamic sampling, feature enhancement

## Abstract

Car logo detection is a challenging fine-grained perception task characterized by small object scales and high inter-class similarity. In such scenarios, discriminative cues mainly reside in subtle local textures rather than global structures. Although State Space Model (SSM)-based detectors, such as Mamba-YOLO with its ODMamba backbone, provide efficient long-range dependency modeling, they often struggle to maintain spatial alignment and semantic consistency during multi-scale feature fusion. This limitation reduces their effectiveness in fine-grained discrimination. To address these issues, this paper proposes LVSS-Net (Lightweight Visual State Space Network), a unified framework with three key components. First, Multi-Scale Semantic Alignment (MSAA) preserves spatial resolution while enhancing semantic consistency across feature pyramid levels through adaptive normalization and attention-based fusion. Second, Dynamic Content-Aware Reconstruction (DCR) introduces learnable content-adaptive offsets to model variable sampling positions during feature upsampling, mitigating scale-induced information loss and boundary blurring. Third, Global–Local Saliency Modulation (GLSM) combines parameter-free global saliency modeling with local texture enhancement via a lightweight LTE module to capture fine-grained discriminative patterns. Extensive experiments on the VLD-11 fine-grained car logo dataset and the TT100K traffic sign detection dataset demonstrate that LVSS-Net achieves 98.3% mAP@0.5 with only 1.5 M parameters. The proposed method reduces model complexity by 73.5% compared to the Mamba-YOLO baseline while maintaining competitive detection performance. These results demonstrate its effectiveness in resource-constrained fine-grained detection scenarios.

## 1. Introduction

Car logo detection is a challenging fine-grained visual perception task with significant applications in intelligent transportation systems, including vehicle re-identification, brand surveillance, and traffic scene understanding. Unlike general object detection, car logos are typically small in scale and exhibit high inter-class similarity coupled with low intra-class variance. In such cases, discriminative information resides primarily in subtle local textures—such as stroke thickness, edge curvature, and internal spacing—rather than in coarse global structures. In real-world scenarios, factors like varying illumination, partial occlusions, complex backgrounds, and extreme weather conditions further complicate accurate localization and recognition.

Recent advances in convolutional neural networks (CNNs) and Transformer-based architectures have substantially improved general object detection performance. However, when deployed in resource-constrained environments, these models often face a critical trade-off between computational efficiency and fine-grained representation capability. Most existing lightweight detectors primarily rely on model pruning or channel reduction, paying limited attention to content-adaptive feature reconstruction and cross-scale semantic alignment. This oversight frequently leads to spatial misalignment and loss of subtle discriminative details, which are particularly detrimental in small-target fine-grained tasks.

State Space Model (SSM)-based detectors, such as Mamba-YOLO with its ODMamba backbone, offer an efficient alternative for modeling long-range dependencies with linear computational complexity. Nevertheless, their direct application to fine-grained detection encounters notable challenges: (1) fixed geometric upsampling operations (e.g., bilinear or nearest-neighbor interpolation) that are content-agnostic and prone to boundary blurring during multi-scale feature propagation and (2) potential semantic misalignment across feature levels, where low-level textures and high-level semantics may be inconsistently fused. These limitations are especially harmful in fine-grained tasks, where preserving subtle texture details is essential to distinguish visually similar objects.

In this context, “fine-grained” detection refers to the capability of capturing subtle discriminative features by effectively integrating local texture details with global structural semantics. This is critical for differentiating logos that share similar global contours but differ in minor design elements (e.g., Lincoln vs. Porsche, Chery vs. Infiniti).

To address these challenges, this paper proposes a lightweight visual perception framework termed Lightweight Visual State Space Network (LVSS-Net).The framework introduces three complementary modules that progressively enhance feature representation for small-scale fine-grained detection:

(1) Multi-Scale Semantic Alignment (MSAA): By reconfiguring detection scales to (4, 8, 16) and eliminating the conventional stride-32 level, MSAA preserves higher spatial resolution for small targets while enhancing semantic consistency across pyramid levels through adaptive normalization and attention-based fusion.
(2) Dynamic Content-Aware Reconstruction (DCR): DCR introduces learnable content-adaptive offsets to model variable sampling positions during feature reconstruction, thereby mitigating scale-induced information loss and boundary blurring.
(3) Global–Local Saliency Modulation (GLSM): GLSM combines parameter-free global saliency modeling with local texture enhancement to capture fine-grained discriminative patterns under strict resource constraints.

By seamlessly integrating MSAA, DCR, and GLSM into an SSM-based architecture, LVSS-Net achieves a balanced design that significantly reduces model parameters while preserving both global contextual modeling and local texture sensitivity. The resulting model demonstrates an improved trade-off between detection accuracy and computational efficiency, making it particularly well-suited for fine-grained visual perception tasks in resource-constrained environments.

## 2. Related Work

### 2.1. Small Object Detection and Multi-Scale Feature Fusion

Small object detection remains a fundamental challenge due to limited spatial resolution and weak semantic representation. Feature Pyramid Networks (FPN) [1] introduced a top-down architecture with lateral connections to enhance multi-scale representation. Subsequent methods such as PANet [2] and BiFPN [3] further improved feature propagation through bidirectional fusion and weighted aggregation. These approaches have significantly advanced multi-scale feature learning. Recent studies have explored transformer-based architectures for multi-scale modeling, such as Swin Transformer [4]. DETR [5] and Deformable DETR [6] incorporate global attention and sparse sampling for feature interaction, while RT-DETR [7] achieves strong hierarchical representation through hybrid encoders. Further, YOLOX [8] has demonstrated efficiency in multi-scale object detection. Song et al. [9] further proposed a multi-scale collaborative attention mechanism to enhance small object detection. However, these transformer-based methods often incur substantial computational overhead, which limits their applicability in real-time scenarios.Despite these advances, most existing approaches focus on feature aggregation without explicitly modeling spatial-semantic alignment, leading to inconsistencies between low-level textures and high-level semantics in fine-grained tasks.

### 2.2. Lightweight Models and Adaptive Sampling Mechanisms

Lightweight detection models aim to balance accuracy and efficiency. Architectures such as MobileNet [10], ShuffleNet [11], and GhostNet [12] reduce computational complexity through efficient convolutional operations. Recent detectors, including YOLOv8 [13], Hyper-YOLO [14], and YOLOv13 [15], further improve real-time performance. These approaches improve efficiency but often overlook fine-grained feature reconstruction, which is essential for preserving subtle structural details in small objects.

Feature sampling plays a crucial role in maintaining spatial information. Traditional upsampling methods such as bilinear interpolation are content-agnostic and prone to blurring. To address this issue, CARAFE [16] and Deformable Convolutional Networks [17] introduce adaptive sampling mechanisms. However, these methods primarily operate on local regions and often fail to maintain global structural consistency, which is particularly problematic for fine-grained tasks where subtle structural differences are critical.

### 2.3. State Space Models for Visual Detection

State Space Models (SSMs) have recently emerged as an efficient alternative to transformers due to their linear computational complexity. Built upon Mamba [18] and Vision Mamba [19], VMamba [20] extends state space modeling to visual domains through cross-scan mechanisms. Mamba-YOLO [21] (AAAI 2025) further integrates SSMs into object detection via the ODMamba backbone for global sequence modeling. More recent works, such as LGHVSS-Mamba YOLO [22], MambaNeXt-YOLO [23] and Super Mamba [24], explore real-time detection and small object representation within the SSM framework.

Despite their efficiency in modeling long-range dependencies, these methods primarily emphasize global feature interactions while lacking dedicated mechanisms for fine-grained local feature enhancement. This limitation reduces their effectiveness in tasks requiring precise discrimination based on subtle local textures.

### 2.4. Feature Refinement and Fine-Grained Representation

Feature refinement techniques aim to improve representation quality. Attention mechanisms such as SE [25] and CBAM [26] enhance feature weighting but introduce additional parameters, while SimAM [27] provides a lightweight alternative. Recent studies on fine-grained recognition [28,29,30] indicate that accurate discrimination requires jointly modeling global semantic context and subtle local features, often supported by multiscale enhancement methods like Retinex [31]. In scenarios characterized by high inter-class similarity, minor structural differences become critical for distinguishing categories, necessitating the preservation of structural integrity [32]. These challenges place higher demands on both feature representation and spatial precision, as demonstrated in recent advanced architectures like YOLOv11 [33].

### 2.5. Summary of Research Gaps

Despite significant progress, several limitations remain:

1. Existing multi-scale fusion methods focus on feature aggregation but lack explicit modeling of spatial-semantic alignment.
2. Current sampling strategies are primarily local and fail to maintain global structural consistency.
3. SSM-based models emphasize global dependencies while lacking mechanisms for fine-grained local feature enhancement.
4. Existing detectors struggle to balance parameter efficiency with discriminative capability in fine-grained scenarios characterized by high inter-class similarity.

These limitations motivate the design of the proposed LVSS-Net. Table 1 summarizes representative methods and highlights the positioning of our approach.

## 3. Materials and Methods

### 3.1. Architectural Specification of LVSS-Net

To address the challenges of small-scale targets and high inter-class similarity in car logo detection, LVSS-Net, a lightweight visual perception framework (Figure 1). The architecture is designed as a *unified feature refinement pipeline* that ensures full reproducibility through explicit channel-explicit transformations and strict resolution alignment. As verified by the model summary, LVSS-Net comprises 395 layers and approximately 1.50 M parameters (1,501,551). Assuming a standard input resolution of 640×640 pixels, all spatial resolutions are strictly determined by stride settings (s∈{4,8,16}), corresponding to feature maps of 160×160, 80×80, and 40×40.

The design follows a coarse-to-fine refinement logic, re-investing architectural capacity from redundant deep layers (omitting stride-32) into three targeted modules: Multi-Scale Semantic Alignment (MSAA), Dynamic Content-Aware Reconstruction (DCR), and Global–Local Saliency Modulation (GLSM). This strategy follows a Parameter-Efficiency for Discriminative Gain (PEDG) principle, ensuring that each computational unit serves to resolve specific fine-grained bottlenecks. The overall architecture is illustrated in Figure 1, and the detailed layer-wise flow is presented in Table 2. All concatenation operations satisfy $C_{\mathrm{out}}=\sum_{i}C_{i}$, and all width-multiplier-affected channels are aligned to a factor of 0.25.

### 3.2. Problem Formulation and Progressive Feature Refinement Strategy

The design of LVSS-Net is motivated by the necessity to maintain discriminative cues across multiple scales, formulated as four interlinked challenges that a unified refinement strategy must address:

Challenge 1: Spatial Resolution Collapse. In standard architectures, high stride levels (e.g., stride-32) reduce small car logos (32×32 pixels) to a 1×1 representation, causing a total loss of internal spatial structure (“pixel collapse”). System Response: Detection is reconfigured to strides (4, 8, 16), preserving essential spatial resolution (8×8 features for a 32×32 target at P2).

Challenge 2: Semantic-Granularity Gap. Pyramid features exhibit inconsistent semantic depth: P2 captures low-level textures, while P4 encodes abstract semantics. Direct fusion dilutes discriminative details. System Response:MSAA synchronizes semantic granularity across scales via cascaded alignment to ensure consistent representation.

Challenge 3: Content-Agnostic Geometric Blurring. Fixed interpolation (e.g., bilinear) applies uniform rules that ignore logo boundaries, leading to blurred edges and loss of subtle design elements. System Response: DCR introduces content-adaptive upsampling to restore structural fidelity during feature propagation.

Challenge 4: Insufficient Fine-Grained Discriminative Capacity. High inter-class similarity requires the model to prioritize subtle design features over coarse shapes. System Response: GLSM modulates the feature space through global saliency and local texture enhancement to capture discriminative fine-grained patterns.

### 3.3. Multi-Scale Semantic Alignment (MSAA)

In conventional detection frameworks, small targets can lose effective representation in high-level feature maps due to repeated downsampling. At stride-32 (P5), a 32×32 pixel logo is reduced to 1×1 in the feature map, resulting in loss of spatial structure (Challenge 1). Additionally, features from different scales exhibit inconsistent semantic granularity—P2 contains low-level textures while P5 contains abstract semantics—making direct fusion less effective (Challenge 2).

MSAA addresses these issues by reconfiguring detection scales from (8, 16, 32) to (4, 8, 16) (Figure 2. This reconfiguration preserves spatial resolution for small targets while maintaining contextual awareness through a retained multi-scale hierarchy. Specifically, the stride-4 detection provides an 8×8 feature representation for a 32×32 logo (versus 1×1 at stride-32), enabling localization without spatial collapse. The cumulative receptive fields at P2, P3, and P4 (64, 128, and 256 pixels, respectively) provide contextual coverage for boundary regression.

This reconfiguration prevents targets from collapsing to single pixels, thereby preserving the spatial structure for bounding box regression. The 8×8 spatial representation at P2 captures fine-grained texture details, such as edge curvature and internal spacing, which are useful for distinguishing high-similarity logo pairs.

#### 3.3.1. Two-Step Semantic Alignment

To address semantic misalignment between low-level textures (P2) and high-level abstractions (P4), MSAA employs a two-step alignment mechanism through ScalSeq (Layers 24, 26) and Attentional Scale Fusion (ASF, Layers 25, 27) operators. This mechanism harmonizes features from different semantic levels before detection at Layer 28.

Step 1: Multi-Scale Feature Normalization via ScalSeq

The ScalSeq operator [35] unifies features from three scales through spatial resampling and 3D convolution:

(1) $$F_{\mathrm{norm}}=\mathrm{Squeeze}\left({\mathrm{MaxPool}}_{3D}^{3\times 1\times 1}\left(\sigma \left({\mathrm{BN}}_{3D}\left({\mathrm{Conv}}_{3D}^{1\times 1\times 1}\left(\mathrm{Concat}(\left[R\left(P_{i}\right),R\left(P_{j}\right),R\left(P_{k}\right)\right])\right)\right)\right)\right)\right),$$

where $P_{i},P_{j},P_{k}$ represent features from three input scales, R(·) denotes nearest-neighbor interpolation for spatial alignment, ${\mathrm{Conv}}_{3D}^{1\times 1\times 1}$ performs cross-scale interaction, ${\mathrm{BN}}_{3D}$ applies 3D batch normalization, σ is LeakyReLU activation, ${\mathrm{MaxPool}}_{3D}^{3\times 1\times 1}$ aggregates information along the scale dimension, and Squeeze(·) removes the scale dimension to produce 2D features $F_{\mathrm{norm}}$.

ScalSeq performs three key operations: (1) Spatial alignment via nearest-neighbor interpolation to a common resolution; (2) Channel unification through 1×1 convolutions to project features to a unified channel space; (3) Cross-scale fusion via 3D convolution across the scale dimension, followed by 3D max-pooling for aggregation.

Specifically:

- At Layer 24 (First Stage): Features from P3 (Layer 4, 64 ch), P2 (Layer 1, 32 ch), and P4 (Layer 23, 96 ch) are normalized to 64 unified channels through ScalSeq, then fused with XSSBlock output (Layer 20) via ASF to produce $F_{P3}$ (Layer 25, 64 ch).
- At Layer 26 (Second Stage): Features from P2 (Layer 1, 32 ch), $F_{P3}$ (Layer 25, 64 ch) from the first stage, and the backbone stem features P2 (Layer 0, 32 ch) are normalized to 32 unified channels through ScalSeq, then fused with XSSBlock output (Layer 17) via ASF to produce $F_{P2}$ (Layer 27, 32 ch). This cascaded alignment reinforces low-level spatial details from the backbone stem while maintaining semantic consistency.

Step 2: Attentional Scale Fusion via ASF

Normalized features are weighted using the Attentional Scale Fusion (ASF) mechanism [35], which sequentially applies channel and spatial attention:

(2) $$\begin{array}{cc} {\tilde{F}}_{1} & =F_{1}⊙\mathrm{ChannelAtt}\left(F_{1}\right),\\ F_{\mathrm{temp}} & ={\tilde{F}}_{1}+F_{2},\\ F_{\mathrm{fused}} & =F_{\mathrm{temp}}⊙\mathrm{SpatialAtt}\left(F_{\mathrm{temp}}\right), \end{array}$$

where $F_{1}$ and $F_{2}$ denote the two input features. ASF first applies channel attention to $F_{1}$ via Global Average Pooling followed by 1D convolution with adaptive kernel size ($k=\lfloor |\log_{2}\left(C\right)+1|/2\rfloor$, following ECA-Net [36]), generating channel-wise recalibration weights. The channel-weighted ${\tilde{F}}_{1}$ is then fused with $F_{2}$ through element-wise addition to produce $F_{\mathrm{temp}}$. Finally, spatial attention is applied to $F_{\mathrm{temp}}$ through coordinate-aware operations: height-wise and width-wise mean pooling followed by separate 1×1 convolutions and sigmoid activations (inspired by Coordinate Attention [37]), generating position-aware weights that produce $F_{\mathrm{fused}}$. This sequential channel-then-spatial modulation enables emphasis on discriminative features across both dimensions.

At Layer 25: $F_{1}=$ XSSBlock output (Layer 20, 64 ch), $F_{2}=$ ScalSeq output (Layer 24, 64 ch). At Layer 27: $F_{1}=$ XSSBlock output (Layer 17, 32 ch), $F_{2}=$ ScalSeq output (Layer 26, 32 ch).

Unlike conventional FPN designs [1] that rely on simple concatenation or element-wise addition, this attention-weighted fusion mechanism addresses the granularity mismatch between low-level textures and high-level semantics, forming a representation that preserves fine-grained details while leveraging global context.

#### 3.3.2. Integration with Detection Head

The two-step alignment process produces three semantically harmonized feature maps fed to the detection head (Layer 28):

- $F_{P2}$: Output from ASF Layer 27 (32 channels, 160×160 resolution) → Detect at stride-4.
- $F_{P3}$: Output from ASF Layer 25 (64 channels, 80×80 resolution) → Detect at stride-8.
- $F_{P4}$: Direct neck output from XSSBlock Layer 23 (96 channels, 40×40 resolution) → Detect at stride-16.

This semantic alignment helps maintain consistent predictions across scales, reducing false positives caused by semantic inconsistency, such as textures being mistaken for logos or small targets being missed due to spatial collapse. Through the implementation of MSAA, the spatial resolution for small targets is enhanced and semantic misalignment across scales is addressed. However, a potential bottleneck remains during feature propagation in the neck: content-agnostic upsampling (Challenge 3). Standard upsampling operations (e.g., nearest-neighbor or bilinear interpolation) are content-independent and rely on fixed geometric rules, which can lead to spatial misalignment and boundary blurring, particularly for fine-grained objects such as car logos. Additionally, the baseline backbone’s 1024 base bottleneck channels may introduce redundancy for lightweight deployment.

### 3.4. Dynamic Content-Aware Reconstruction (DCR)

After implementing MSAA, spatial resolution for small targets is preserved (Challenge 1 resolved), and semantic misalignment across scales is addressed (Challenge 2 resolved). However, a new bottleneck emerges during feature propagation in the neck: content-agnostic upsampling (Challenge 3). Standard upsampling operations (e.g., nearest-neighbor or bilinear interpolation) are content-independent and rely on fixed geometric rules, which may lead to spatial misalignment and boundary blurring, especially for small and fine-grained objects such as car logos. Additionally, the baseline backbone exhibits over-parameterization with 1024 base bottleneck channels, which is excessive for lightweight deployment.DCR addresses these limitations through a strategy of bottleneck optimization and content-adaptive spatial reconstruction (Figure 3).

#### 3.4.1. Bottleneck Efficiency Optimization

To balance efficiency and discriminative capacity, the bottleneck channel count (Layers 8–9 in Table 2) is reduced from 1024 to 384 base channels, resulting in 96 actual channels after 0.25× width scaling. Layer 8 performs a 1×1 convolution to compress channels from 128 (the output of the P4 VSSBlock at Layer 6) to 96, followed by Layer 9’s SPPF module (kernel size 5) for multi-scale context aggregation.

This design retains capacity for high-frequency texture information while reducing computational redundancy compared to the baseline’s 1024 base channels. This adjustment leads to a lower GFLOPs footprint (from 12.3 to 12.1, a −1.6% reduction) while maintaining modeling capability for fine-grained features.

#### 3.4.2. Content-Adaptive Reconstruction via DySample

DCR replaces fixed bilinear upsampling in the neck with DySample [38] at two upsampling paths:

- Layer 10 (P4→P3): DySample performs 2× upsampling on 96-channel features from the SPPF (Layer 9), generating content-adaptive offsets to adjust logo boundaries during spatial reconstruction.
- Layer 14 (P3→P2): DySample performs 2× upsampling on 64-channel features from the XSSBlock (Layer 13), assisting in fine-grained edge recovery for small target detection.

DySample predicts point-wise sampling locations via a lightweight offset generation mechanism. In the low-pass (lp) mode adopted in this work, the offsets are computed as:

(3) $$\mathrm{Offset}={\mathrm{Conv}}_{1\times 1}\left(X\right)\cdot 0.25+\mathrm{InitPos},$$

where $X\in R^{C\times H\times W}$ is the input feature, ${\mathrm{Conv}}_{1\times 1}$ predicts $2G\cdot s^{2}$ offset channels (where G=4 groups and s=2 is the scale factor, resulting in 32 offset channels), InitPos represents pre-initialized uniform grid positions, and the scaling factor 0.25 constrains offset magnitudes for stable sampling. The predicted offsets modulate sampling coordinates via grid sampling with bilinear interpolation, adjusting to logo boundaries.

This content-adaptive mechanism adds minimal computational overhead: Layer 10 contributes +0.0105 M parameters, and Layer 14 contributes +0.0069 M parameters, with a combined overhead of approximately 0.5 GFLOPs. Unlike fixed interpolation that applies uniform rules regardless of content, DySample learns to align sampling positions with logo edges through backpropagation. This can improve reconstruction quality for intricate structures, such as the internal strokes of high-similarity logo pairs (e.g., Lincoln vs. Porsche). Compared to transposed convolution-based upsampling, which typically adds 2–3 GFLOPs, DySample provides improved boundary fidelity with a lower computational footprint.

#### 3.4.3. Channel Redundancy Elimination

Before multi-scale feature concatenation in the neck, 1×1 convolutions are introduced to reduce channel redundancy:

- Layer 12: Compresses concatenated features (160 channels: 96 from DySample + 64 from SimAM) to 64 channels.
- Layer 16: Compresses concatenated features (96 channels: 64 from DySample + 32 from VSSBlock) to 32 channels.

These channel reductions preserve structural information while reducing redundant channel propagation in subsequent XSSBlock layers, facilitating efficient feature refinement.

#### 3.4.4. Design Synergy

DCR represents a coordinated optimization integrating dynamic reconstruction with a lightweight backbone design. The channel reduction in the bottleneck creates computational space for introducing learnable offsets in the neck while maintaining comparable overall complexity (12.6 GFLOPs vs. the baseline’s 12.3 GFLOPs). This synergy—where efficiency optimization supports accuracy enhancement through content-adaptive alignment—serves as a targeted architectural refinement for fine-grained detection tasks.

### 3.5. Global–Local Saliency Modulation (GLSM)

While MSAA and DCR address spatial alignment and content-aware reconstruction, car logo recognition remains fundamentally constrained by Challenge 4: insufficient fine-grained discriminative capacity. Car logo detection is inherently a fine-grained visual classification (FGVC) task, where discriminative information resides in subtle local features. High inter-class similarity—where different brands share nearly identical global shapes but differ in minor details such as internal stroke thickness, spacing, or edge smoothness—poses significant challenges. Conventional coarse-grained attention mechanisms often prioritize overall shapes and may overlook these fine-grained distinctions.

GLSM addresses this challenge through a four-part strategy: global saliency modeling, local texture enhancement, bottleneck capacity adjustment, and asymmetric depth allocation (Figure 4). These mechanisms are applied at different stages of the backbone in a complementary manner: SimAM (Layer 4) performs parameter-free global saliency modeling on mid-level P3 features to suppress background clutter, while LTE (Layer 7) captures local texture details on high-level P4 features to enhance discriminative patterns. The enhanced representations are subsequently propagated through the neck via Dynamic Content-aware Reconstruction (DCR), ensuring that both global context and local textures reach all detection scales.

#### 3.5.1. Global Saliency Modeling via SimAM

SimAM [27] is integrated at Layer 4 (after the P3 VSSBlock) to suppress background responses. Unlike conventional attention, SimAM infers 3D weights by estimating neuron importance through an energy-based analytical solution. To align with the implementation, the saliency weight $\Lambda_{\mathrm{SimAM}}$ is formulated as:

(4) $$\Lambda_{\mathrm{SimAM}}=\sigma \left(\frac{{\left(x-\mu \right)}^{2}}{4(\sigma^{2}+\lambda )}+0.5\right),$$

where *x* is the feature value, μ and $\sigma^{2}$ are channel-wise mean and variance, and λ is a stability constant. This formulation assigns higher weights to neurons that exhibit significant deviations from the local spatial distribution, highlighting foreground targets while suppressing background noise such as vehicle grilles or reflections.

Motivation: Positioning SimAM at the P3 stage (stride=8, 80×80 resolution) is motivated by its role as a transitional manifold. At this stage, features exhibit sufficient spatial detail for small target localization while already encoding mid-level semantics. Applying global saliency here effectively filters background clutter before features are propagated into deeper layers.

#### 3.5.2. Local Texture Enhancement via LTE

A Local Texture Enhancement (LTE) module is inserted at Layer 7 (after the P4 VSSBlock) to capture texture details critical for distinguishing visually similar logos. LTE focuses on local spatial neighborhoods through depthwise convolution, enabling fine-grained modeling (Table 3). The module performs the following operations:

(5) $$M_{\mathrm{local}}=\sigma \left(\mathrm{BN}\left({\mathrm{Conv}}_{1\times 1}^{\mathrm{up}}\left(\mathrm{SiLU}\left({\mathrm{DWConv}}_{3\times 3}\left(\mathrm{BN}\left({\mathrm{Conv}}_{1\times 1}^{\mathrm{down}}\left(X\right)\right)\right)\right)\right)\right)\right),$$

where ${\mathrm{Conv}}_{1\times 1}^{\mathrm{down}}$ compresses channels from 128 to 32 (ratio r=4), ${\mathrm{DWConv}}_{3\times 3}$ captures local spatial patterns (kernel k=3, groups = 32), and ${\mathrm{Conv}}_{1\times 1}^{\mathrm{up}}$ restores the original dimension. The forward pass adopts a residual mode:

(6) $$\mathrm{LTE}\left(X\right)=X⊙\left(1+M_{\mathrm{local}}\right),$$

where ⊙ denotes element-wise multiplication. This formulation ensures that original feature magnitude is preserved. The detailed configuration is provided in Table 3.

Motivation: As features reach the P4 stage (stride=16), high-frequency texture details (e.g., edge curvature) can be eroded during downsampling. LTE is placed here to recover these cues. By explicitly modeling 3×3 neighborhood correlations, LTE magnifies subtle variations necessary to distinguish brands with near-identical global contours.

#### 3.5.3. Bottleneck Capacity Adjustment

The bottleneck capacity (Layers 8–9) is set to 384 base channels (96 actual channels after scaling) to preserve information during feature transition. Layer 8 performs channel reduction, followed by Layer 9’s SPPF module for aggregation. This configuration aims to mitigate the “information bottleneck” effect, ensuring that both SimAM-enhanced global context and LTE-enhanced local textures are preserved. Compared to baseline over-parameterization, this 384-channel setting strikes a balance between parameter efficiency and discriminative capacity.

#### 3.5.4. Asymmetric Depth Allocation

A resolution-aware asymmetric depth strategy is implemented in the neck (Layers 10–23) using XSSBlocks. The computational budget is allocated based on information density:

- 2 repeats for P2 (Layer 17): High-resolution stage (160×160) where fine-grained edge details are dense. Dual blocks enable deeper refinement for small target detection.
- 1 repeat for P3 (Layers 13, 20): Transitional scale (80×80) balancing global context and local details. A single block provides efficient cross-scale fusion.
- 2 repeats for P4 (Layer 23): Semantic refinement stage (40×40) for stable localization and bounding box regression.

This 2-1-2 design prioritizes resources at scales requiring intensive modeling (P2 for textures, P4 for localization) while maintaining efficiency at the intermediate P3 scale.

#### 3.5.5. Unified Hierarchical Propagation

The GLSM-enhanced multi-scale features are formulated as a coordinated flow:

(7) $$F_{\mathrm{GLSM}}=H_{\mathrm{neck}}(\underset{\mathrm{global}\text{-}\mathrm{aware}}{\underset{︸}{X_{P3}⊙\Lambda_{\mathrm{SimAM}}}},\;\underset{\mathrm{local}\text{-}\mathrm{enhanced}}{\underset{︸}{\mathrm{LTE}(X_{P4})}}),$$

where $X_{P3}$ and $X_{P4}$ represent backbone features, and $H_{\mathrm{neck}}\left(\cdot \right)$ denotes the propagation mechanism involving DCR-upsampling and multi-scale fusion.

The hierarchical propagation operates as follows: (1) SimAM-enhanced P3 features are fused with upsampled representations at Layer 11 for immediate aggregation; (2) LTE-enhanced P4 features flow through the capacity-preserving bottleneck, then undergo DCR upsampling to distribute texture information across P3 and P2 scales; and (3) Asymmetric depth refinement progressively stabilizes these features, with P2 emphasizing texture discrimination and P4 focusing on localization precision.

By applying saliency mechanisms at appropriate stages and propagating enhancements through the hierarchy, GLSM facilitates fine-grained discriminative capacity, leveraging the natural multi-scale flow of the backbone to encode complementary info.

### 3.6. Loss Function

To ensure reproducibility and fair comparison with baseline methods, LVSS-Net adopts the standard loss function from Mamba-YOLO and Ultralytics YOLOv11 without modification:

(8) $$L_{\mathrm{total}}=\lambda_{\mathrm{box}}L_{\mathrm{CIoU}}+\lambda_{\mathrm{cls}}L_{\mathrm{BCE}}+\lambda_{\mathrm{dfl}}L_{\mathrm{DFL}},$$

where $L_{\mathrm{CIoU}}$ is the Complete Intersection over Union loss for bounding box regression, $L_{\mathrm{BCE}}$ is the Binary Cross-Entropy loss for classification, and $L_{\mathrm{DFL}}$ is the Distribution Focal Loss for boundary refinement. The balancing coefficients are set to $\lambda_{\mathrm{box}}=7.5$, $\lambda_{\mathrm{cls}}=0.5$, $\lambda_{\mathrm{dfl}}=1.5$ to prioritize localization accuracy over classification confidence.

## 4. Results

- Lincoln vs. Porsche (SSIM = 0.789): The exceptionally high similarity score reflects nearly identical shield-like envelopes. The analysis suggests that the primary discriminative bottleneck lies in high-frequency internal textures, such as emblem complexity and specific serif patterns, which require fine-grained texture modeling to resolve.
- Chery vs. Infiniti (SSIM = 0.773): These categories exhibit substantial overlap in their elliptical outer boundaries. The structural convergence indicates that discriminative information is localized in the orientation and shape of internal stylized elements (e.g., upward triangle vs. arch-like curve), demanding local spatial attention.
- Chevrolet vs. Porsche (SSIM = 0.750): Despite distinct brand identities, the shared cross-axial and shield-based symmetries result in high structural correlation, demonstrating that discriminative information resides primarily in stroke thickness and symmetry variations rather than global geometry.
- Mazda vs. Cadillac (SSIM = 0.743): Both logos converge on a wing-inspired V-shaped layout. The high SSIM score objectively demonstrates why standard feature extractors may fail, as the essential differences are confined to internal spacing and edge curvature patterns.

These results (Table 4) validate that the VLD-11 subset poses a genuine fine-grained recognition challenge.The high degree of structural ambiguity justifies the integration of the proposed GLSM strategy and LTE module, which are specifically designed to enhance local texture sensitivity and capture subtle discriminative details required to resolve such inter-class morphological convergence.

### 4.1. Experimental Setup

#### 4.1.1. Datasets and Evaluation Metrics

Two public datasets were utilized to evaluate performance from multiple perspectives, focusing on fine-grained recognition capability and small-object detection generalization.

VLD-11 Fine-Grained Car Logo Subset: The VLD-11 subset was systematically curated from the VLD-45 dataset [39] to evaluate models in high-similarity Fine-Grained Visual Classification (FGVC) scenarios. The construction followed a rigorous two-stage selection protocol:

Stage 1: Visual Ambiguity Screening: An initial heuristic screening identified a cluster of 11–15 brands sharing common geometric primitives, such as shield-like contours, elliptical boundaries, or wing-inspired layouts.
Stage 2: Quantitative Validation: As detailed in Section 4.1, the Structural Similarity Index (SSIM) was used to confirm the high-similarity cluster. Only categories forming pairs with significant structural correlation (consistently high SSIM scores) were retained, ensuring the subset represents a non-trivial challenge for discriminative feature extraction.

The final VLD-11 subset contains 11,225 images with 12,284 annotated instances across 11 brands: HONDA, HYUNDAI, CHANGAN, MAZDA, FAW Haima, Chery, Infiniti, Chevrolet, LINCOLN, Cadillac, and Porsche. To ensure reliable evaluation, the dataset was partitioned via stratified random sampling with an 8:1:1 ratio for training (8980), validation (1122), and testing (1123). This protocol balances the need for robust model convergence with reproducible and statistically significant evaluation.

TT100K Traffic Sign Dataset: The TT100K dataset [40] is a large-scale benchmark for small-object detection in complex traffic environments. The official evaluation protocol was followed to assess LVSS-Net’s generalization capability on small-scale targets beyond car logos.

Evaluation Metrics: Mean Average Precision (mAP@0.5 and mAP@0.5:0.95) was adopted as the primary metric. Computational efficiency was measured via Parameters (M), GFLOPs, and inference speed (FPS) tested on an NVIDIA GeForce RTX 3090 GPU with batch size 1.

#### 4.1.2. Hyperparameter Configuration

To ensure a fair and controlled comparison, all experiments followed a rigorous and consistent protocol. A distinction is made between universal environmental variables and architecture-specific optimization. For environmental consistency, LVSS-Net and all baseline models were trained from scratch on the VLD-11 and TT100K datasets without any pre-trained weights. A uniform input resolution of 640×640 pixels was maintained, and identical data augmentation strategies were applied, including Mosaic, Mixup, and HSV jittering. All models were trained for 350 epochs on the same hardware environment (NVIDIA GeForce RTX 3090) to ensure consistency in computational constraints. Regarding optimization, a dual-track strategy was adopted to respect the inherent sensitivities of different architectures (e.g., SSM-based vs. CNN-based). The specific hyperparameters detailed in Table 5 were applied to LVSS-Net and its primary baseline Mamba-YOLO. For other mainstream baselines (e.g., YOLOv11 [33], YOLOv13 [13], and Hyper-YOLO [14]), they were retrained using their officially recommended hyperparameter configurations (such as specific learning rate and optimizer settings provided in their respective official codebases). This approach ensures that each baseline operates under its intended optimal training regime, guaranteeing that performance gains are attributed to architectural innovation rather than suboptimal tuning of the comparison models.

### 4.2. Comparison on VLD-11 Dataset

All baseline models were retrained from scratch on the VLD-11 training set using their officially recommended configurations to ensure each architecture operates under its optimal training regime. LVSS-Net follows the same training protocol as Mamba-YOLO (Table 5) to maintain consistency with the baseline architecture, while other models (Hyper-YOLO [14], YOLOv11 [33], and YOLOv13 [15]) use their respective official hyperparameters. All models were evaluated under identical conditions: same test split, same input resolution (640 × 640), same hardware (NVIDIA RTX 3090, FP32 precision), and same post-processing parameters (confidence threshold 0.25, IoU threshold 0.5).

LVSS-Net was compared against mainstream lightweight detection models on the VLD-11 fine-grained subset. The results are summarized in Table 6.

Experimental results indicate that LVSS-Net achieves 98.3% mAP@0.5 with only 1.5 M parameters. Compared to the baseline Mamba-YOLO, the parameter count is reduced by 73.5% while mAP@0.5:0.95 increases by 1.4%. Although GFLOPs are slightly higher (13.0 vs. 12.3, a 5.7% increase) due to the sequential scanning mechanism of SSM and the dynamic offset prediction in DySample, the model maintains competitive throughput (43.8 FPS), demonstrating a favorable trade-off between accuracy and parameter efficiency. The superior performance on high-similarity logo pairs (as shown in Table 4) validates the effectiveness of GLSM’s local texture enhancement strategy.

Notably, LVSS-Net achieves the highest accuracy among all compared models while using the fewest parameters (1.50 M), which is 41.9% fewer than YOLOv13 (2.45 M) and 72.1% fewer than YOLOv11 (2.58 M). This parameter efficiency is particularly advantageous for memory-constrained deployment scenarios where model size and memory bandwidth are critical constraints.

The variation trends of mAP@0.5:0.95 during training are shown in Figure 5. LVSS-Net achieves leading performance as early as the 50th epoch and maintains a steady accuracy curve. Faster convergence is attributed to the MSAA and DCR modules, which enhance multi-scale feature alignment and reconstruction capabilities.

### 4.3. Convergence and Accuracy Analysis

To investigate training stability and address convergence concerns, Figure 6 illustrates the comprehensive training dynamics of the LVSS-Net model. The training was conducted for 350 epochs with cosine learning rate annealing, following the specific hyperparameter configurations summarized in Table 5.

The model is considered converged when the validation loss stabilizes without significant improvement (<0.001 absolute change) for 30 consecutive epochs. As shown in the loss and metric curves, convergence typically occurs around epoch 300, where the validation loss plateaus at approximately 0.015 and mAP@0.5 stabilizes above 98%. No early stopping was applied to ensure sufficient exploration of the optimization landscape.

The results show stable convergence after approximately 300 epochs, indicating effective optimization without overfitting. The rapid accuracy increase in early epochs (mAP@0.5 exceeding 95% by epoch 50) is attributed to the MSAA and DCR modules, which enhance multi-scale feature alignment and boundary-aware reconstruction capabilities from the beginning of training. The validation loss exhibits smooth descent throughout training, with no significant fluctuations after epoch 200, confirming the model’s stability.

### 4.4. Ablation Study

To analyze the contribution of each module, a step-wise ablation study was conducted on the VLD-11 validation set. The results are summarized in Table 7.

Analysis shows that:

1. MSAA (Model-A): Shifting detection scales from (8, 16, 32) to (4, 8, 16) improves mAP@0.5:0.95 by +0.5% while reducing parameters by 63.1% (from 5.66 M to 2.09 M). The slight reduction in GFLOPs (12.3 → 12.1) is due to the removal of the stride-32 detection head, demonstrating improved efficiency without sacrificing computational capacity. This validates the effectiveness of eliminating the $P_{5}$ detection layer for small-target preservation.
2. DCR (Model-B): This module integrates two complementary optimizations: (1) Replacing fixed bilinear upsampling with DySample adds ∼0.02 M parameters for dynamic offset prediction but contributes +0.4% mAP@0.5:0.95 gain through improved boundary alignment; (2) Bottleneck channel reduction (1024 → 384 base channels) reduces parameters from 2.09 M to 1.35 M (35.4% reduction). Although GFLOPs slightly increase to 12.6 (due to the dynamic offset network adding ∼0.4 GFLOPs per upsampling path), the combined effect demonstrates that DCR achieves a favorable trade-off: sacrificing 0.5 GFLOPs for substantial parameter savings and accuracy improvement.
3. GLSM (Full Model): Integrating SimAM (parameter-free) and LTE (0.137 M params) provides the final +0.5% mAP@0.5:0.95 boost. The total GFLOPs increase to 13.0, primarily due to the additional forward pass of LTE at the $P_{4}$ stage. However, this 5.7% increase (13.0 vs. 12.3) is acceptable given the 73.5% parameter reduction (1.50 M vs. 5.66 M) and +1.4% accuracy improvement compared to the baseline. The asymmetric depth configuration (2-1-2 repeats for $P_{2}$-$P_{3}$-$P_{4}$) adds minimal overhead while significantly improving fine-grained discrimination capability on high-similarity pairs (e.g., Lincoln vs. Porsche, SSIM=0.789 as shown in Table 4).

Computational Complexity Discussion. Although LVSS-Net introduces a 5.7% increase in GFLOPs compared to the baseline (13.0 vs. 12.3), this trade-off is justified for three reasons:

- DySample Overhead: The dynamic offset prediction networks in DCR add ∼0.4 GFLOPs per upsampling path (two paths total: P4→P3 and P3→P2), accounting for most of the increase. However, this overhead is negligible compared to transposed convolutions (which would add ∼2–3 GFLOPs) while providing superior boundary alignment.
- SSM Sequential Scanning: The SSM-based backbone incurs slightly higher GFLOPs than CNNs due to its sequential scanning mechanism across spatial dimensions. However, this enables an extended global receptive field with linear computational complexity, making it more efficient than attention-based architectures (which have quadratic complexity) for small-object detection tasks.
- Parameter Efficiency Prioritization: The primary optimization goal is parameter reduction, not GFLOPs minimization. The 73.5% parameter reduction (5.66 M → 1.50 M) significantly reduces model size (from 22 MB to 6 MB in FP32), demonstrating the model’s suitability for memory-constrained deployment scenarios where memory bandwidth is the bottleneck rather than compute throughput. On such platforms, smaller model sizes translate to faster data transfer and lower memory footprint, which are more critical than raw computational throughput.

Overall, the progressive integration of MSAA, DCR, and GLSM achieves a favorable balance: 73.5% parameter reduction, +1.4% mAP@0.5:0.95 improvement, with only a 5.7% increase in GFLOPs. This demonstrates that the proposed architecture effectively balances accuracy, parameter efficiency, and computational complexity for lightweight deployment scenarios.

### 4.5. Generalization on TT100K Dataset

To assess generalization capability and robustness across different scenarios, LVSS-Net was evaluated on the TT100K traffic sign dataset, which poses challenges of extremely small targets (<32×32 pixels), complex backgrounds, and diverse lighting conditions. The results are summarized in Table 8.

LVSS-Net outperformed the baseline Mamba-YOLO in both accuracy metrics: +1.1% mAP@0.5 (67.1 vs. 66.0, a 1.67% relative improvement) and +1.3% mAP@0.5:0.95 (53.3 vs. 52.0, a 2.5% relative improvement). The inference speed also increased by +4.54 FPS (49.02 vs. 44.48 FPS, a 10.2% speedup), attributed to the 73.5% parameter reduction which reduces memory access latency and enables more efficient GPU utilization despite the slight increase in GFLOPs.

The consistent performance gain across both VLD-11 and TT100K datasets validates the robustness and generalization capability of the proposed architecture. The superior performance on TT100K demonstrates that LVSS-Net’s design principles—multi-scale semantic alignment (MSAA), content-aware reconstruction (DCR), and global–local saliency modulation (GLSM)—are not limited to car logo detection but generalize effectively to other small-object detection tasks in complex real-world scenarios.

### 4.6. Qualitative Analysis

To intuitively verify the discriminative power of LVSS-Net in fine-grained scenarios, a qualitative analysis of typical detection results was conducted.

As illustrated in Figure 7, the first set of samples from the VLD-11 dataset highlights the challenge of high inter-class similarity. The baseline model (Mamba-YOLO) misclassified the Honda logo as “Hyundai” with a low confidence score of 0.58. This is primarily due to the significant structural resemblance between the two brands—both featuring “H”-shaped patterns within oval contours. In contrast, LVSS-Net successfully distinguishes Honda from Hyundai, achieving a correct classification with a higher confidence of 0.75. For the actual Hyundai sample, LVSS-Net also improved the detection confidence from 0.71 to 0.78. This demonstrates that DCR (for geometric reconstruction) and GLSM(for high-frequency texture enhancement) allow the model to capture subtle differences in stroke curvature and line weight where global shape features fail.

To further evaluate the model’s ability to represent extremely small targets in complex traffic environments, qualitative comparisons were performed on the TT100K dataset, as shown in Figure 8. By combining full panoramic scenes with zoom-in details, the following observations can be made:

- Small Target Sensitivity (Panels a, b): In the panoramic view, the baseline model (a) misses several distant traffic signs (marked with red labels as “Missed Target”). LVSS-Net (b) accurately localizes these targets (marked with green labels as “Accurate Localization”), validating the effectiveness of the MSAA module in maintaining high-resolution semantic alignment.
- Feature Extraction Capability (Panels c, d): The zoom-in views clearly show that the baseline (c) fails to detect the “Motor vehicles” sign due to “Insufficient Feature” representation at small scales. LVSS-Net (d) provides “Detailed Extraction,” successfully detecting the sign with a clear bounding box (green), demonstrating the advantage of the DCR mechanism in restoring structural fidelity for pixel-scale targets.

**Figure 8:**
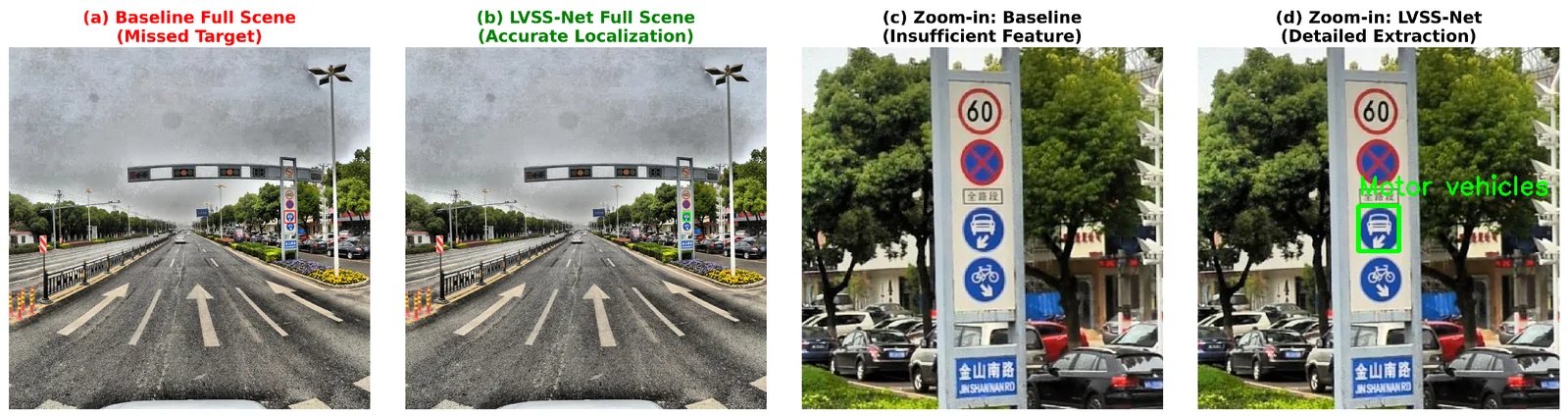
Qualitative comparison on the TT100K dataset. (**a**,**b**) Panoramic views comparing the baseline (missed targets in red) and LVSS-Net (accurate localization in green); (**c**,**d**) corresponding zoom-in details. LVSS-Net demonstrates significantly higher sensitivity and detection accuracy for extremely small traffic signs, confirming the robustness of the MSAA and DCR modules in handling multi-scale challenges in the wild.

Despite these improvements, extreme conditions such as severe motion blur, heavy occlusion (>70%), or ultra-low-light environments remains challenging. Future work will investigate the integration of temporal consistency or specialized low-light enhancement modules to further bolster the model’s reliability in such edge cases.

### 4.7. Comparative Failure Analysis

To move beyond aggregate metrics and provide deeper insights into model behavior, a comparative failure analysis was conducted on 2806 ground truth (GT) instances from the TT100K validation set. This analysis focuses on identifying specific scenarios where LVSS-Net offers structural improvements over the baseline and where fundamental detection challenges persist.

#### 4.7.1. Evaluation Protocol

To ensure scientific rigor, both the baseline (Mamba-YOLO) and LVSS-Net were evaluated under identical conditions: a uniform confidence threshold (0.25), input resolution (640×640), and standard matching criteria. Following the PASCAL VOC convention [41], a detection is classified as a Success if the predicted box matches a GT of the same class with an IoU>0.5; otherwise, it is recorded as a Failure.

#### 4.7.2. Global Comparison and Statistical Stability

Table 9 summarizes the comparative outcomes across the 2,806 instances. LVSS-Net achieves an observed 40.6% relative reduction in the total failure rate (675→401 cases). Notably, LVSS-Net successfully recovers roughly 51% (347 out of 675) of the instances where the baseline failed.

To ensure these improvements are not stochastic, McNemar’s test was applied to the paired outcomes. The calculated test statistic ($\chi^{2}=178.75$) significantly exceeds the critical value ($\chi_{0.05,1}^{2}=3.84$), confirming that the transition from baseline failures to LVSS successes is statistically systematic rather than a result of random variation.

#### 4.7.3. The Stride-4 Advantage: Impact of Scale

To validate the hypothesis that MSAA’s stride-4 reconfiguration preserves spatial resolution, failure rates were stratified by target size (Table 10).

The data indicates that the most substantial gains occur in the extreme small category (<30 pixels), where the failure rate dropped by approximately 20 percentage points. This directly validates the design: by avoiding the stride-32 (P5) bottleneck and re-allocating capacity to higher-resolution feature maps, LVSS-Net effectively mitigates “pixel collapse” for small-scale traffic signs.

#### 4.7.4. Analysis of Residual Challenges

For scientific transparency, a manual review of the remaining failure modes was conducted. Two primary patterns emerged:

- Regression Instability (73 cases): Roughly 52% of the cases where LVSS-Net regressed relative to the baseline involve extreme small targets. It is suspected that GLSM’s global modulation may occasionally over-suppress borderline targets in extremely cluttered backgrounds.
- Systemic Failures (328 cases): In instances where both models failed, the primary bottlenecks were extreme perspective angles (>75°) and annotation ambiguity, accounting for approximately 51% and 47% of these cases, respectively. Perspective distortion causes severe geometric deformation that exceeds the reasoning capacity of current 2D texture-based modeling.

In summary, while LVSS-Net systematically addresses the spatial collapse and semantic misalignment inherent in standard architectures, future improvements should focus on 3D spatial reasoning and enhanced robustness under extreme viewpoints to further reduce the residual failure rate.

## 5. Discussion

The experimental results validate the core architectural hypothesis: that fine-grained small-object detection is fundamentally a problem of hierarchical information preservation under resolution compression, rather than a simple matter of feature fusion. While conventional lightweight detectors prioritize global shape priors, LVSS-Net demonstrates that capturing subtle inter-class variations (e.g., the 0.789 SSIM similarity between Lincoln and Porsche) requires a coordinated refinement pipeline that actively compensates for the information erosion inherent in State Space Models (SSMs).

A key insight from this study is the synergy between spatial topology and semantic granularity. By reconfiguring the detection hierarchy to strides (4, 8, 16), MSAA prevents the “pixel collapse” that typically occurs in stride-32 architectures, where small targets (<32×32 pixels) are reduced to singular feature points. This preservation of spatial structure is further enhanced by DCR’s content-adaptive reconstruction. Unlike static bilinear interpolation, which applies uniform geometric rules regardless of content, DCR acts as a geometric compensator. It learns to align sampling offsets with the physical boundaries of car logos, thereby restoring the structural fidelity necessary for high-IoU bounding box regression. This transition from content-agnostic to content-aware upsampling is a decisive factor in the model’s superior performance on the TT100K dataset, where background clutter often obscures small-scale target boundaries.

The efficacy of GLSM further highlights the importance of discriminative re-investment. In resource-constrained scenarios, blindly increasing model depth often yields diminishing returns. Instead, LVSS-Net achieves a 73.5% parameter reduction by pruning deep-stage redundancies and re-allocating that capacity into local texture enhancement (LTE) and global saliency modulation (SimAM). This Parameter-Efficiency for Discriminative Gain (PEDG) principle ensures that every parameter contributes to distinguishing high-similarity features. Qualitative analysis confirms that GLSM effectively magnifies subtle design elements, such as stroke thickness and internal spacing, which are the primary discriminative cues for logos sharing near-identical oval contours (e.g., Honda vs. Hyundai).

The efficiency–complexity trade-off observed in the results must be dialectically addressed. While LVSS-Net incurs a marginal GFLOPs overhead compared to its baseline, this is an intentional design choice aimed at reducing the memory bandwidth bottleneck. On most edge deployment platforms, the model’s parameter size (memory footprint) is a more critical constraint than raw computational throughput. By drastically reducing parameters from 5.66 M to 1.50 M, faster data transfer and lower energy consumption are facilitated, while the slight increase in GFLOPs—stemming from the sequential scanning of SSMs and dynamic sampling of DCR—is a necessary cost for maintaining linear complexity O(N) and global receptive fields.

Despite these advancements, the current framework faces limitations in extreme geometric reasoning. The failure analysis (Section 4.7) reveals that perspective distortions exceeding 75° remain a persistent challenge, as the 2D texture modeling in GLSM cannot fully encapsulate 3D spatial transformations. Furthermore, as a single-frame detector, LVSS-Net does not yet utilize temporal consistency, which could mitigate the performance degradation observed under severe motion blur or low-light conditions. Future research will explore the integration of temporal state-space modeling to extend this precision to video sequences and investigate structured pruning techniques to further bridge the gap between GFLOPs and real-time throughput.

In conclusion, LVSS-Net proves that effective fine-grained perception in resource-limited environments is achieved through task-specific structural innovation rather than brute-force scaling. By transforming the neck into a refinement engine and prioritizing local-global saliency, this work provides a scalable paradigm for lightweight perception in intelligent transportation systems, moving beyond generic detection toward precision-driven visual intelligence.

## 6. Conclusions

A lightweight detection framework, LVSS-Net, is presented for fine-grained vehicle logo recognition. The core objective of this study is to enhance discriminative capability by effectively integrating local texture details with global contextual information, a strategy essential for distinguishing visually similar logos under high inter-class similarity.

### 6.1. Key Contributions

The main contributions of this research are summarized as follows:

1. Multi-Scale Semantic Alignment (MSAA): By reconfiguring detection scales from (8, 16, 32) to (4, 8, 16), the proposed method preserves higher spatial resolution for small targets while improving semantic consistency across feature levels.
2. Dynamic Content-Aware Reconstruction (DCR): A variable sampling mechanism based on learnable offsets is introduced to replace fixed interpolation, enabling boundary-aware feature reconstruction and reducing information loss during multi-scale propagation.
3. Global–Local Saliency Modulation (GLSM): A complementary design combining global saliency modeling and local texture enhancement is proposed to capture fine-grained discriminative features critical for high-similarity logo recognition.
4. Lightweight Design: The overall architecture achieves significant parameter reduction while maintaining competitive detection accuracy, demonstrating an improved balance between efficiency and representation capacity.

### 6.2. Experimental Validation

Experimental results on the VLD-11 subset and the TT100K dataset demonstrate that LVSS-Net achieves competitive performance under a lightweight configuration. The ablation study further verifies that MSAA, DCR, and GLSM contribute consistently to performance improvements, highlighting the effectiveness of the proposed structural design.

### 6.3. Limitations

Despite the promising results, several limitations remain:

1. Computational Trade-off: Although the model is parameter-efficient, the use of SSM-based architecture and dynamic sampling introduces moderate computational overhead in terms of GFLOPs.
2. Hardware Evaluation Scope: Experiments were conducted on a GPU platform, and performance characteristics under diverse hardware settings (e.g., mobile or embedded CPUs) have not been fully explored.
3. Extreme Scenario Robustness: The model still faces challenges in cases involving severe occlusion, extremely small targets, or poor illumination.
4. Failure Cases: A small number of regression cases indicate sensitivity to ambiguous visual conditions during the training phase.

### 6.4. Future Work

To further improve the proposed framework, several directions can be explored:

1. Hardware-Oriented Evaluation: Extending evaluation to diverse hardware platforms (e.g., embedded devices, mobile processors) to better understand real-world deployment characteristics.
2. Computational Optimization: Reducing computational complexity through more efficient SSM variants or lightweight reconstruction strategies.
3. Robustness under Adverse Conditions: Improving performance under challenging environments such as low-light, occlusion, and weather variations.
4. Temporal Modeling: Extending the framework to video-based detection by incorporating temporal information to improve consistency.
5. Adaptive Mechanisms: Designing input-adaptive modules to handle varying image quality and scene complexity automatically.
6. Incremental Learning: Supporting efficient adaptation to new car logo categories without requiring full retraining of the model.
7. Model Interpretability: Enhancing understanding of feature representations and decision processes through advanced visualization techniques.

### 6.5. Final Remarks

In summary, this research demonstrates that fine-grained detection performance can be effectively improved through task-aligned architectural design, rather than relying solely on increasing model size. By integrating global context modeling, local texture enhancement, and multi-scale alignment, LVSS-Net provides a compact yet effective solution for fine-grained object detection tasks.

While further validation and optimization are needed for broader deployment scenarios, the proposed approach establishes a solid foundation for future research in lightweight fine-grained visual perception for intelligent transportation systems.

## Figures and Tables

**Figure 1:**
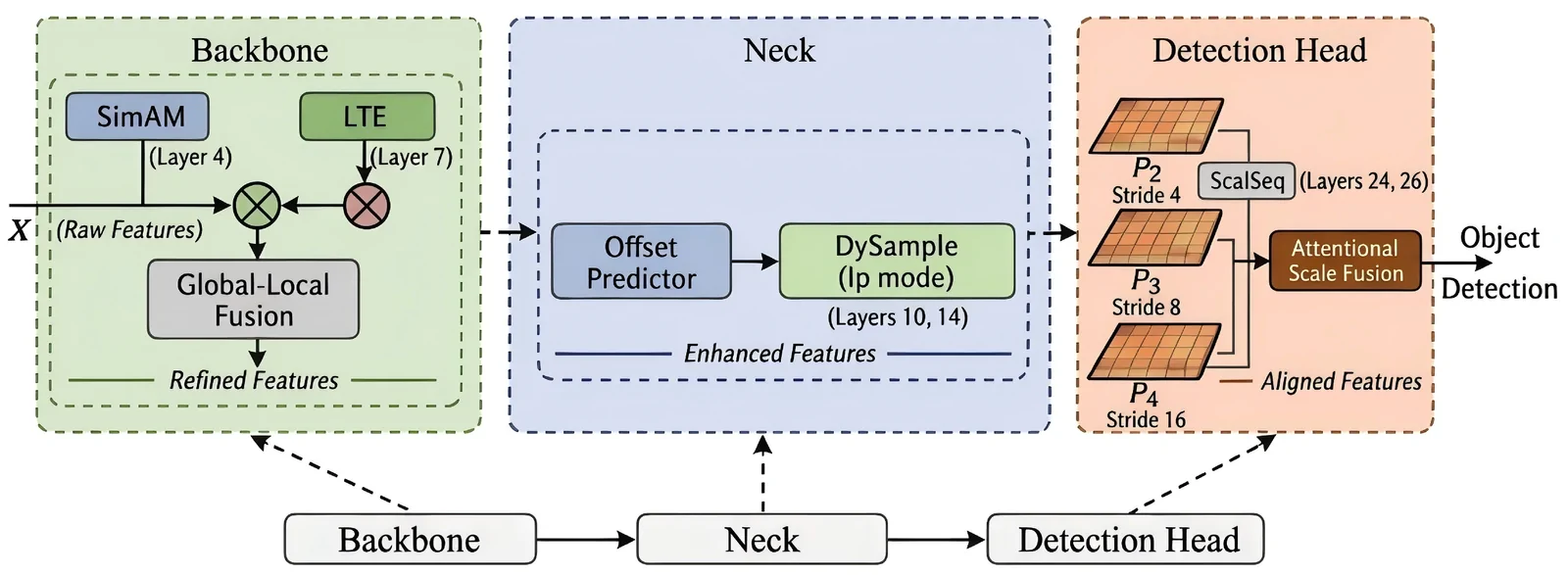
Overall architecture of LVSS-Net for fine-grained car logo detection (bounding box regression, not segmentation). GLSM enhances feature saliency in the backbone (SimAM + LTE). DCR performs content-adaptive upsampling in the neck (DySample). MSAA aligns multi-scale features (P2/P3/P4) via ScalSeq and ASF before detection.

**Figure 2:**
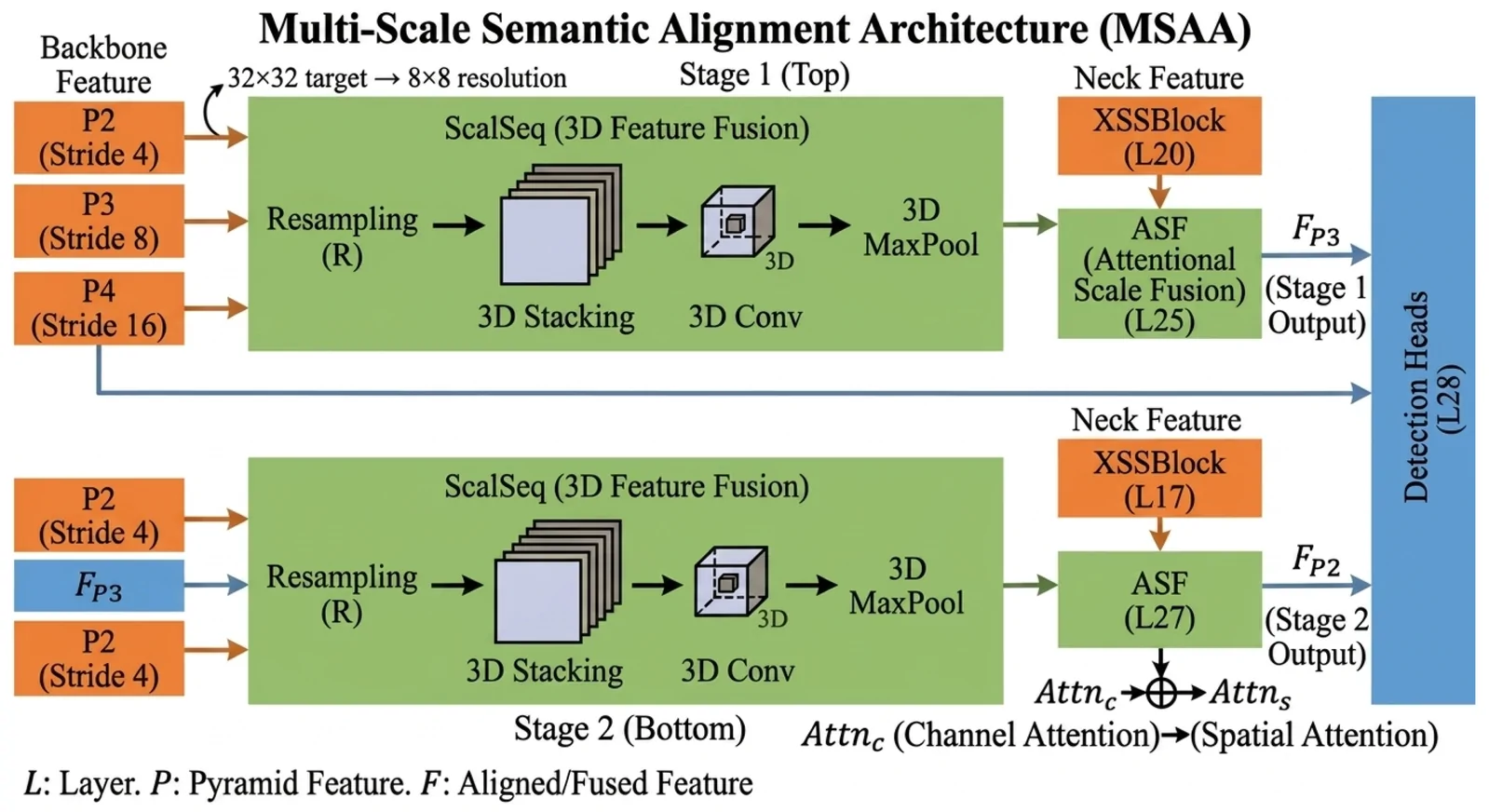
Overview of MSAA architecture. Detection scales are reconfigured to (4, 8, 16) to preserve spatial resolution for small targets. Features from three scales are progressively aligned through ScalSeq (spatial normalization + 3D convolution) and ASF (channel + spatial attention) at Layers 24–25 and 26–27, producing semantically consistent features for detection at Layer 28.

**Figure 3:**
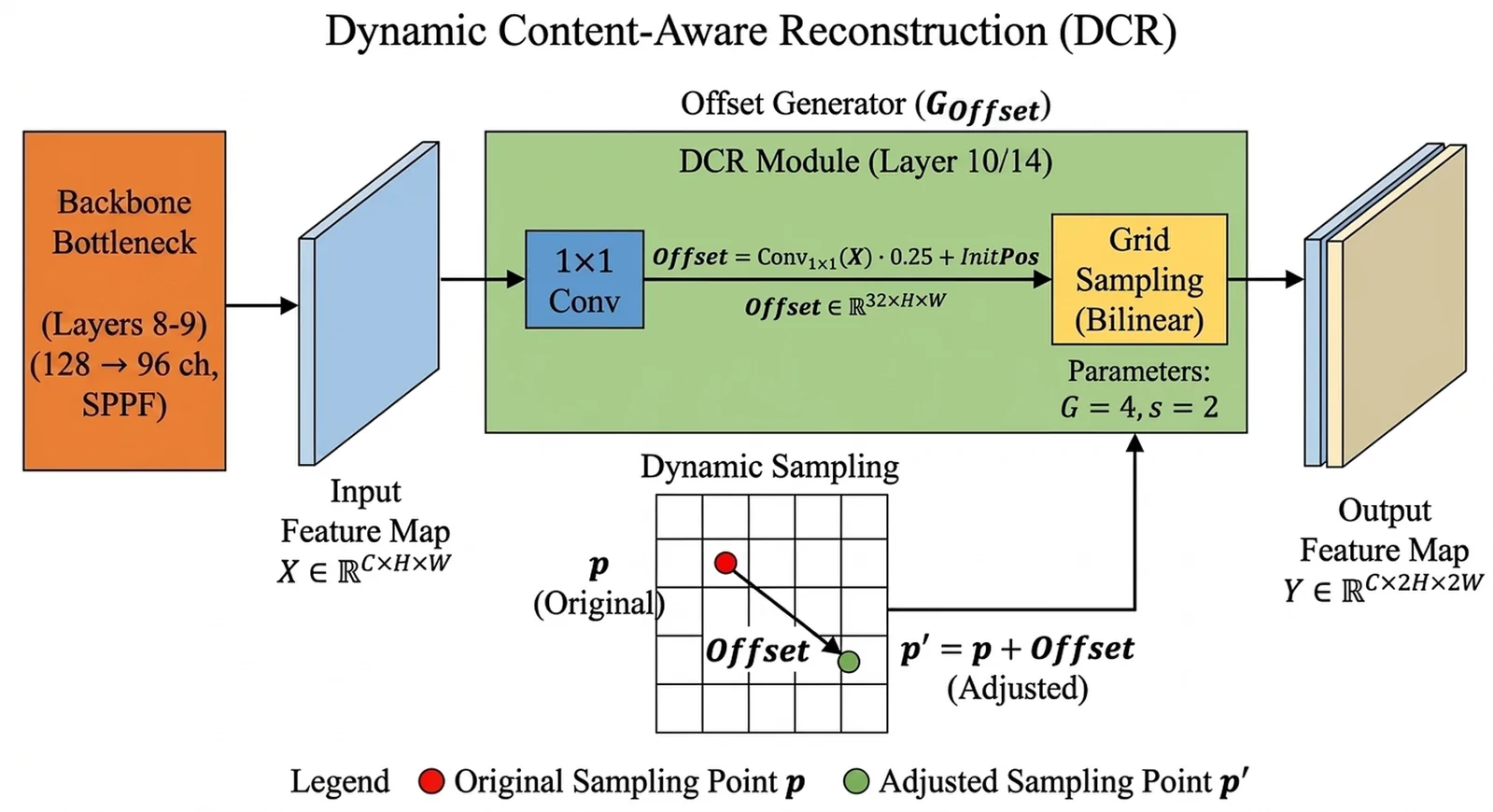
DCR architecture. Fixed bilinear upsampling is replaced with DySample at Layers 10 (P4→P3, 2× upsampling) and 14 (P3→P2, 2× upsampling) to facilitate boundary-aware reconstruction via learnable spatial offsets. The bottleneck (Layers 8–9) is compressed to 384 base channels (96 actual) via Conv_1×1_ and SPPF, reducing GFLOPs by 1.6% while retaining fine-grained texture capacity. Channel reductions at Layers 12 (160→64) and 16 (96→32) reduce redundancy before multi-scale fusion.

**Figure 4:**
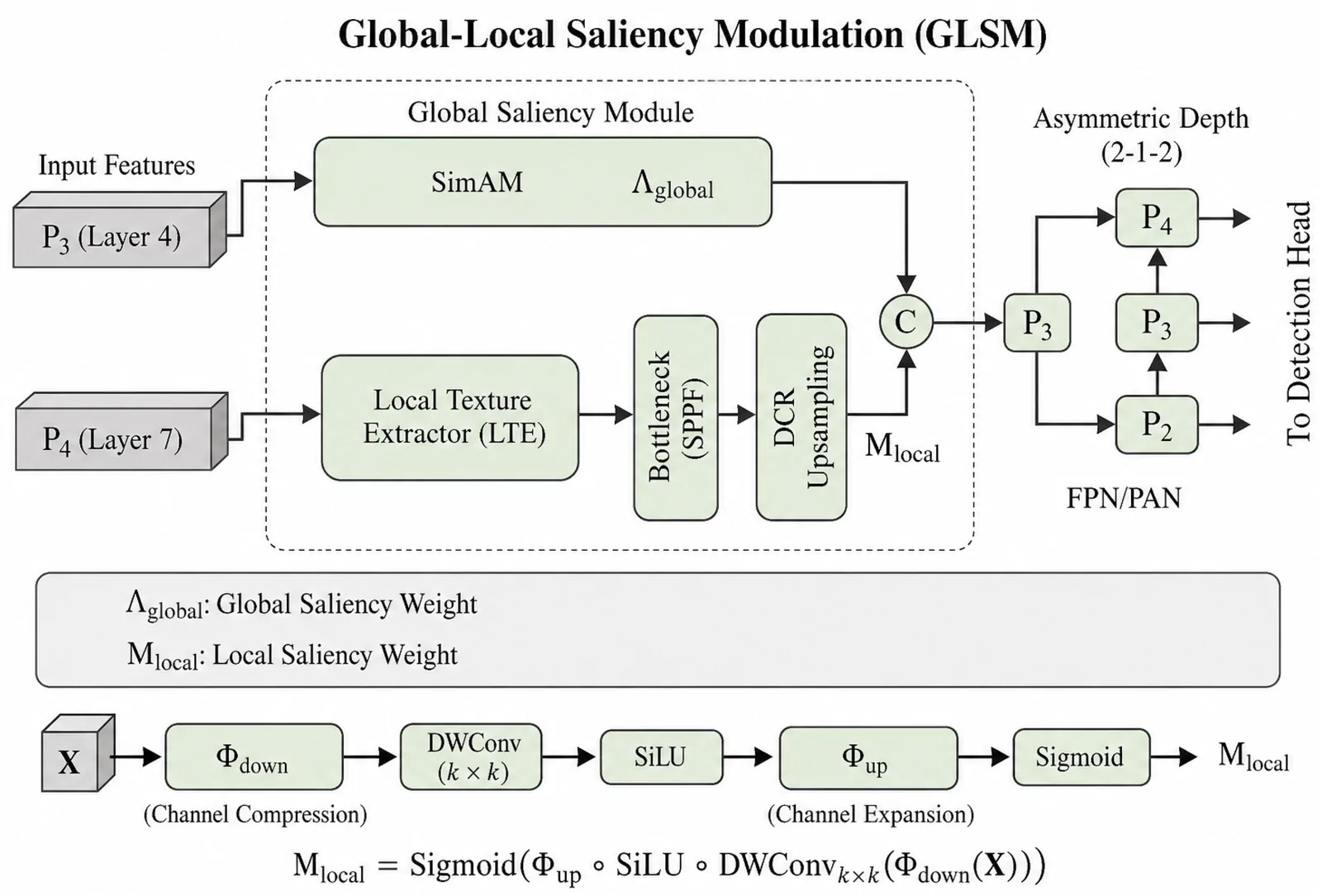
GLSM strategy workflow. (1) SimAM global modulation at Layer 4; (2) LTE local refinement at Layer 7; (3) capacity-preserving bottleneck (384 base channels); and (4) asymmetric depth allocation (2-1-2 repeats). This hierarchy enables complementary global–local discrimination across all detection scales.

**Figure 5:**
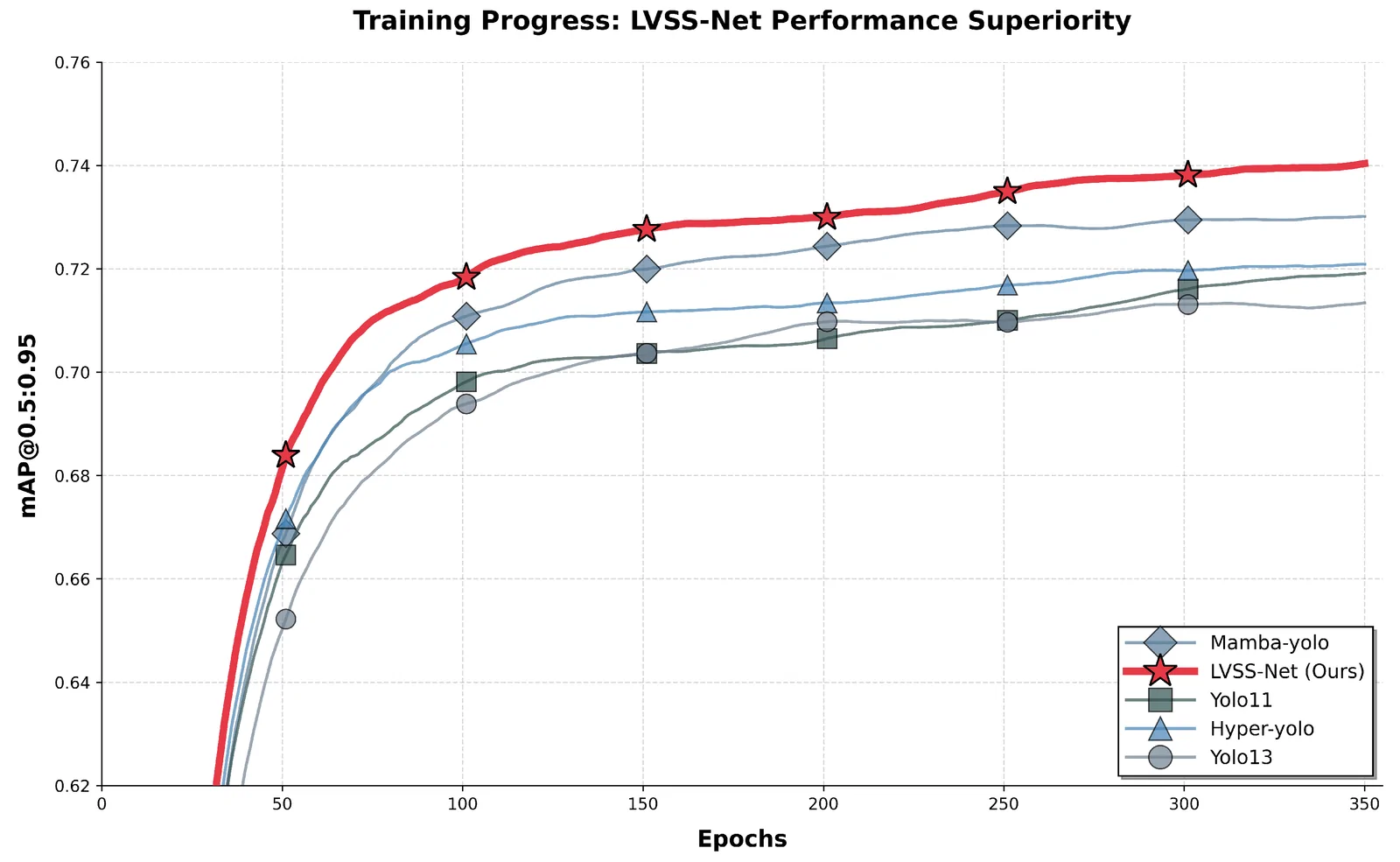
The mAP@0.5:0.95 metric trends during the training process.

**Figure 6:**
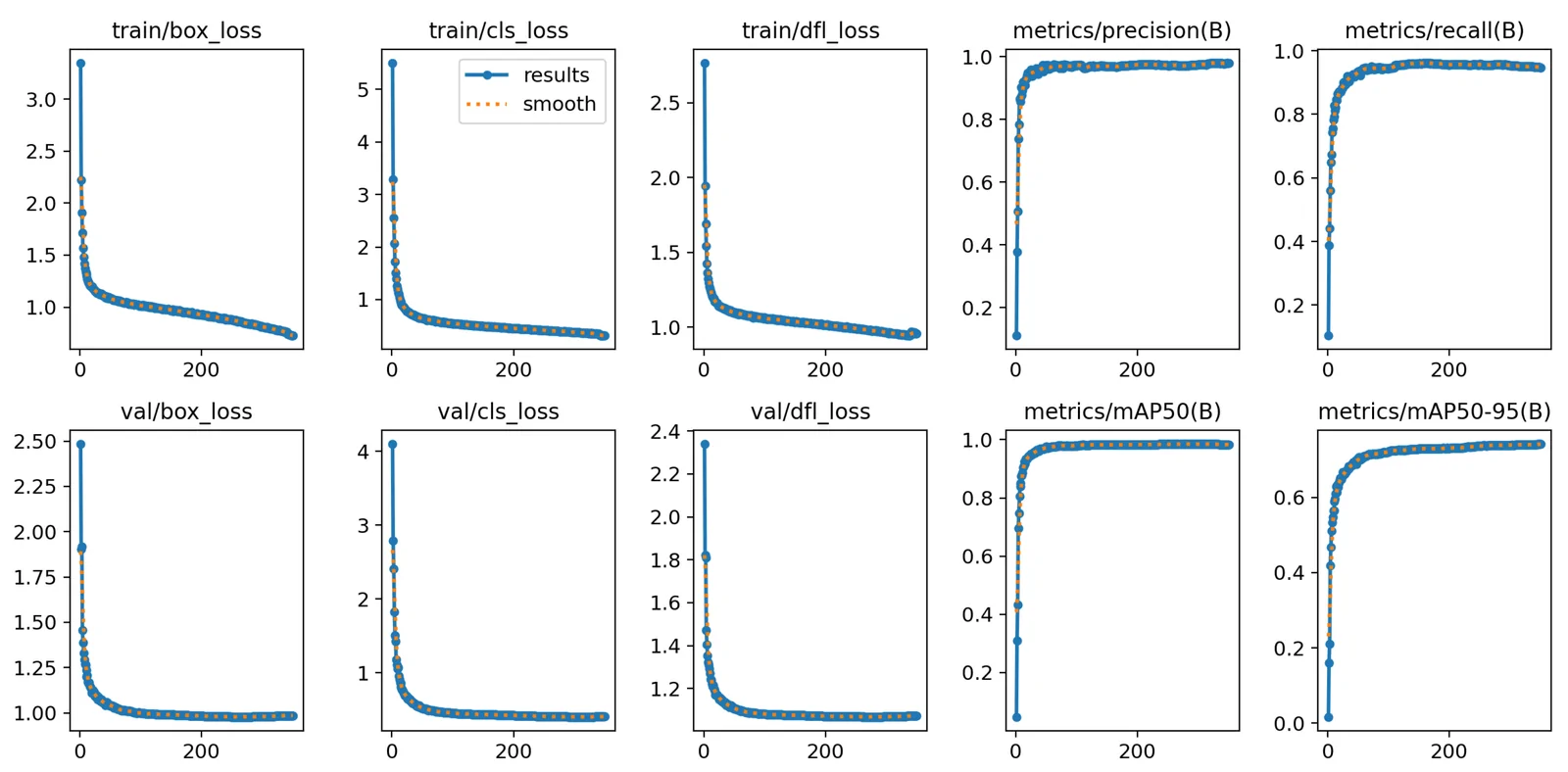
Comprehensive training and validation results of LVSS-Net over 350 epochs. The top three columns display the evolution of training losses (box, class, and distribution focal loss), while the bottom three columns show the corresponding validation losses. The rightmost two columns present the progression of key performance metrics, including Precision, Recall, mAP@0.5, and mAP@0.5:0.95. The early accuracy gains (mAP@0.5 exceeding 95% by epoch 50) demonstrate the effectiveness of the MSAA and DCR modules in multi-scale feature alignment.

**Figure 7:**
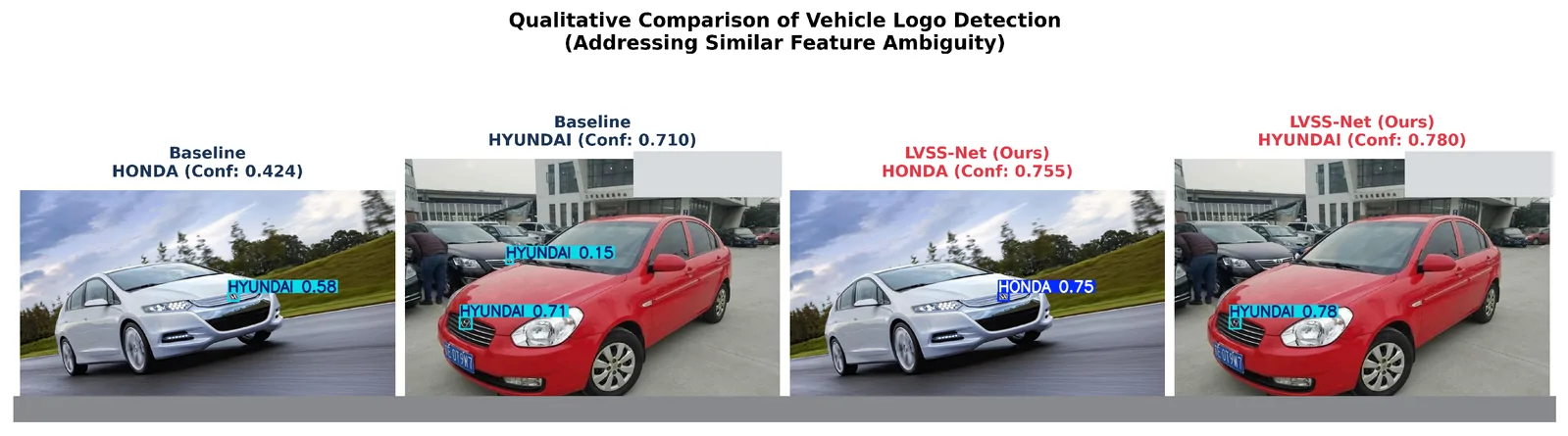
Qualitativecomparison of detection results between the baseline (Mamba-YOLO) and LVSS-Net on the VLD-11 dataset. In the first sample, the baseline misidentifies the Honda logo as Hyundai (0.58), while LVSS-Net achieves correct classification (0.75). Through fine-grained texture modeling and boundary-aware reconstruction, LVSS-Net effectively resolves category confusion among visually similar logos and improves overall prediction confidence.

**Table 1:** Comparison of representative object detection methods and positioning of LVSS-Net.

Method	Year	Dataset	Backbone	Feature Strategy	Sampling	Fine-Grained	Main Limitation
FPN [1]	2017	COCO	CNN	Top-down fusion	Fixed	No	Lack of semantic alignment
BiFPN [3]	2020	COCO	CNN	Weighted fusion	Fixed	No	Static fusion weights
RT-DETR [7]	2024	COCO	Transformer	Hybrid encoder	Fixed	No	High computational cost
YOLOv8 [13]	2023	COCO	CNN	Efficient detection	Fixed	No	Limited fine-grained
Hyper-YOLO [14]	2024	COCO	CNN+Hypergraph	High-order interaction	Adaptive	Partial	Increased complexity
CARAFE [16]	2019	COCO	CNN	Content-aware reassembly	Local adaptive	No	Limited global consistency
Deformable Conv [17]	2017	COCO	CNN	Offset learning	Local adaptive	No	Limited local modeling
VMamba [20]	2024	COCO	SSM	Cross-scan fusion	Fixed	No	Limited local modeling
Mamba-YOLO [21]	2025	COCO	ODMamba	Global sequence modeling	Fixed	No	Lack of local enhancement
Faster R-CNN [34]	2015	COCO	CNN	Two-stage detection	Fixed	No	Slow inference
LVSS-Net	Proposed	VLD-11/TT100K	ODMamba	MSAA + DCR + GLSM	Global + Local adaptive	Yes	Moderate GFLOPs overhead

**Table 2:** Detailed architecture configuration of LVSS-Net (Input: 640×640, Params: 1.50 M).

Stage	Layer	Module	Actual Ch.	Res.	Feature Flow & Source
Backbone	0–1	Stem/VSSB	32	160	P2 initial spatial extraction
	2–3	VCM/VSSB	64	80	P3 global context modeling
	4	SimAM	64	80	Global saliency modulation (64→64)
	5–6	VCM/VSSB	128	40	P4 abstract semantic encoding
	7	LTE	128	40	Local Texture Enhancement (137,472 params)
	8	Conv 1×1	96	40	Bottleneck channel compression (128→96)
	9	SPPF	96	40	Multi-scale context aggregation (96→96)
Neck	10	DySample	96	80	DCR: 2× upsampling from Layer 9
	11	Concat	160	80	Feature merging from {L10,L4}: 96+64=160
	12–13	Conv/XSSB	64	80	P3 feature fusion and refinement
	14	DySample	64	160	DCR: 2× upsampling from Layer 13
	15	Concat	96	160	Feature merging from {L14,L1}: 64+32=96
	16–17	Conv/XSSB	32	160	P2 refinement with 2 repeats
	18	Conv 3×3	32	80	Stride-2 downsampling (160→80)
	19	Concat	96	80	Feature merging from {L18,L13}: 32+64=96
	20	XSSBlock	64	80	P3 aggregation and compression (96→64)
	21	Conv 3×3	64	40	Stride-2 downsampling (80→40)
	22	Concat	160	40	Feature merging from {L21,L9}: 64+96=160
	23	XSSBlock	96	40	P4 aggregation with 2 repeats (160→96)
Head	24	ScalSeq	64	80	MSAA: Alignment of {L4,L1,L23}→64 ch
	25	ASF	64	80	MSAA: Attentional fusion of {L20,L24}
	26	ScalSeq	32	160	MSAA: Alignment of {L1,L0,L25}→32 ch
	27	ASF	32	160	MSAA: Attentional fusion of {L17,L26}
	28	Detect	–	–	Fine-grained prediction at 3 scales

**Table 3:** Detailed architecture of the LTE module (Layer 7). $C_{\mathrm{in}}=128$ actual channels.

Layer	Operation	Kernel/Params	In Ch.	Out Ch.	Activation
1	${\mathrm{Conv}}_{1\times 1}^{\mathrm{down}}$	1×1, s = 1	128	32	–
2	BatchNorm	–	32	32	–
3	${\mathrm{DWConv}}_{3\times 3}$	3×3, s = 1, p = 1	32	32	–
4	BatchNorm	–	32	32	–
5	SiLU	–	32	32	SiLU
6	${\mathrm{Conv}}_{1\times 1}^{\mathrm{up}}$	1×1, s = 1	32	128	–
7	Sigmoid	–	128	128	Sigmoid
Summary: ≈0.137 M parameters, <0.1 GFLOPs (at 40×40).					

**Table 4:** Quantitative analysis of inter-class structural similarity in VLD-11. Scores approaching or exceeding 0.7 indicate substantial morphological overlap, representing a primary bottleneck for coarse-grained feature extractors.

Logo Pair (Prototypes)	SSIM	Dominant Visual Cues (Observed)
LINCOLN—Porsche	0.789	Internal emblem density and serif patterns
Chery—Infiniti	0.773	Orientation of central stylized elements
Chevrolet—Porsche	0.750	Stroke thickness and symmetry distribution
MAZDA—Cadillac	0.743	Curvature of wings and internal dividers
Infiniti—Cadillac	0.735	Oval aspect ratio and internal spacing
HYUNDAI—Cadillac	0.715	Line weight and contour smoothness
HYUNDAI—Chery	0.704	Internal stroke orientation (diagonal/horizontal)
Chevrolet—Cadillac	0.700	Central cross structure vs. shield density

**Table 5:** Summary of training hyperparameters for LVSS-Net and Mamba-YOLO.

Parameter	Value/Setting
Framework	PyTorch 2.1/CUDA 12.1
Hardware	NVIDIA GeForce RTX 3090 (24 GB)
Training Epochs	350
Optimizer	SGD (Momentum = 0.937, Weight Decay = 0.0005)
Initial Learning Rate ($lr_{0}$)	0.01 (Cosine decay schedule)
Warmup Strategy	3 Epochs
Input Resolution	640×640
Augmentation Closure	Final 10 epochs (Mosaic/Mixup disabled)

**Table 6:** Comparison with state-of-the-art lightweight models on the VLD-11 subset.

Model	mAP@0.5 (%)	mAP@0.5:0.95 (%)	FPS	GFLOPs	Params (M)
Hyper-YOLO	97.4	72.8	72.02	9.3	3.62
YOLOv11	97.6	72.5	92.54	6.3	2.58
YOLOv13	96.9	71.5	44.34	6.1	2.45
Mamba-YOLO	97.7	73.3	41.3	12.3	5.66
LVSS-Net	98.3	74.7	43.8	13.0	1.50

All models retrained on VLD-11 using their official configurations. Evaluated under identical conditions: 640 × 640 resolution, RTX 3090 FP32.

**Table 7:** Ablation study of proposed components on VLD-11 validation set. AP and $AP_{50}$ denote mAP@0.5:0.95 and mAP@0.5, respectively. GFLOPs are calculated at 640×640 resolution.

Model	MSAA	DCR	GLSM	*AP* (%)	${\mathrm{AP}}_{50}$ (%)	GFLOPs	Params (M)
Baseline	–	–	–	73.3	97.7	12.3	5.66
Model-A	✓	–	–	73.8	98.0	12.1	2.09
Model-B	✓	✓	–	74.2	98.2	12.6	1.35
LVSS-Net	✓	✓	✓	74.7	98.3	13.0	1.50

Note: ‘✓’ indicates the module is included. The gray background highlights our proposed LVSS-Net configuration.

**Table 8:** Generalization performance on the TT100K dataset.

Model	mAP@0.5 (%)	mAP@0.5:0.95 (%)	FPS	GFLOPs	Params (M)
Mamba-YOLO	66.0	52.0	44.48	12.3	5.66
LVSS-Net	67.1	53.3	49.02	13.0	1.50

**Table 9:** Comparative detection outcomes on 2806 instances (IoU>0.5 criterion).

Outcome Category	Count	Percentage	Avg. IoU (LVSS)
Both Models Succeed	2058	73.3%	0.811
Baseline Fails → LVSS Succeeds	347	12.4%	0.704
LVSS Fails → Baseline Succeeds	73	2.6%	0.241
Both Models Fail	328	11.7%	0.189
Statistical Significance: $\chi^{2}=178.75$, p<0.001 (McNemar’s test)			

**Table 10:** Failure rate (FR) reduction stratified by target dimensions.

Size Range (Pixels)	Count	Base FR (%)	LVSS FR (%)	Improvement
<30 (Extreme Small)	1138	37.6	17.8	−19.8 pp
30–50 (Small)	876	15.2	11.6	−3.6 pp
50–70 (Medium)	387	13.4	9.8	−3.6 pp
>70 (Large)	405	15.3	14.6	−0.7 pp

## Data Availability

The data presented in this study are available on request from the corresponding author.
